# Anti-tumor NAMPT inhibitor, KPT-9274, mediates gender-dependent murine anemia and nephrotoxicity by regulating SIRT3-mediated SOD deacetylation

**DOI:** 10.1186/s13045-021-01107-0

**Published:** 2021-06-29

**Authors:** Shaneice Mitchell, Pu Zhang, Matthew Cannon, Larry Beaver, Amy Lehman, Bonnie Harrington, Deepa Sampath, John C. Byrd, Rosa Lapalombella

**Affiliations:** 1 Division of Hematology, The Ohio State University, 455 OSUCCC, 410 West 12th Avenue, Columbus, OH 43210 USA; 2 Department of Pathology, Stanford University, Palo Alto, CA USA; 3 College of Pharmacy, The Ohio State University, Columbus, OH USA; 4 Center for Biostatistics, Department of Bioinformatics, The Ohio State University, Columbus, OH USA; 5 College of Veterinary Medicine, Michigan State University, Lansing, MI USA

**Keywords:** NAMPT, Leukemia, Erythropoietin, Niacin, SOD

## Abstract

KPT-9274 is a phase 1 first-in-class dual PAK4/NAMPT inhibitor for solid tumor and non-Hodgkin’s lymphoma. It demonstrates pre-clinical efficacy toward a broad spectrum of acute myeloid leukemia (AML) subtypes by inhibiting NAMPT-dependent NAD^+^ production. NAMPT is the rate-limiting enzyme in the salvage metabolic pathway leading to NAD^+^ generation. Tumor cells which are deficient in de novo pathway enzyme NAPRT1 are addicted to NAMPT. In clinical trials, treatment with NAMPT inhibitors resulted in dose-limiting toxicities. In order to dissect the mechanism of toxicity, mice were treated with KPT-9274 and resulting toxicities were characterized histopathologically and biochemically. KPT-9274 treatment caused gender-dependent stomach and kidney injuries and anemia. Female mice treated with KPT-9274 had EPO deficiency and associated impaired erythropoiesis. KPT-9274 treatment suppressed SIRT3 expression and concomitantly upregulated acetyl-manganese superoxide dismutase (MnSOD) in IMCD3 cells, providing a mechanistic basis for observed kidney toxicity. Importantly, niacin supplementation mitigated KPT-9274-caused kidney injury and EPO deficiency without affecting its efficacy. Altogether, our study delineated the mechanism of KPT-9274-mediated toxicity and sheds light onto developing strategies to improve the tolerability of this important anti-AML inhibitor.

**Supplementary Information:**

The online version contains supplementary material available at 10.1186/s13045-021-01107-0.

**To the Editor**,

The therapeutic potential of targeting NAMPT, an NAD^+^ biosynthetic enzyme, has been demonstrated in several cancers. Several NAMPT inhibitors have entered phase I trials to date (e.g. FK866, GMX1777, and KPT-9274) [1–4]. However dose-limiting toxicities such as thrombocytopenia and gastrointestinal (GI) toxicities have been observed. While the preclinical toxicity profile of KPT-9274 (the first orally bioavailable NAMPT inhibitor) [5, 6] recapitulates the expected class level GI and hematopoietic toxicities clinically [5], the preclinical retinal or cardiac toxicities, seen with other NAMPT inhibitors [7], were not observed. Though KPT-9274 treatment did not impair leukocyte viability in vitro, reduced red blood cell counts in patients given KPT-9274 clinically has been reported [4]. Mitigating any potential toxicity and improving the tolerability are unmet needs for this class of inhibitors. Herein, we conducted in vivo studies to dissect the potential side effects and underlying mechanisms.

Tumor-free mice were treated with a therapeutically effective dose of KPT-9274 (150 mg/kg, p.o.) or the vehicle once daily. Mild cell death/loss of gastric epithelial cells (Fig. 1B) and tubular epithelial injury, as evident by cortical collapse and interstitial fibrosis in kidneys (Fig. 1C), were observed in the KPT-9274-treated group with no noticeable injuries in the small intestine and eyes. Lesions were much more severe in female mice (Fig. 1C) with robust renal cell death as evidence by TUNEL-positive regions (Fig. 1D). Interestingly, blood-urea-nitrogen (BUN) levels in KPT-9274-treated female NRGS mice were significantly higher than those of vehicle-treated counterparts, while in both genders of NSG mice, they are comparable between treatment groups (Fig. 1E). KPT-9274 treatment did not alter creatinine levels and BUN/creatinine ratios in both NSG and NRGS mice (Fig. 1E and Additional file 1: Figure S1). KPT-9274 treatment didn’t alter serum potassium and chloride levels in both genders, but reduced sodium levels in female mice (Additional file 1: Table 1).

**Fig. 1:**
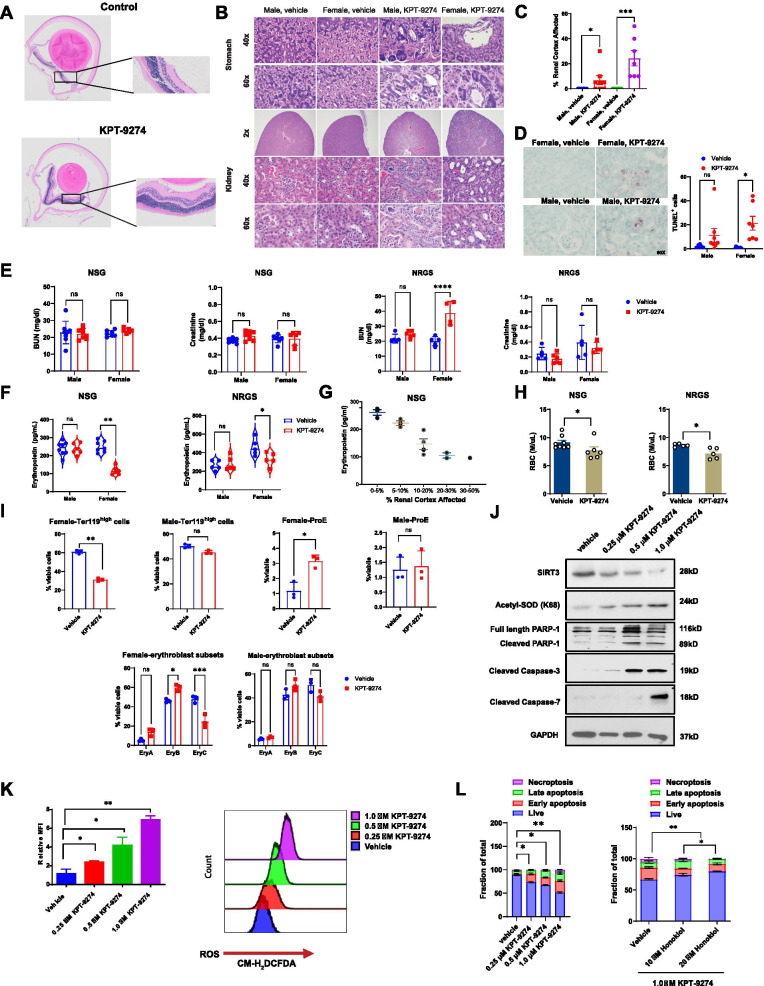
KPT-9274 causes gender-dependent kidney toxicity and anemia. **A** 4× and 20× representative images of vehicle control-treated and KPT-9274-treated NSG mouse eyes showing no disruption of retinal epithelial and neuronal cell layers. **B** Histopathology of stomachs and kidneys of KPT-9274-treated and vehicle-treated NSG mice (representative images at 2× , 40× and 60× magnifications). There is evidence of nephrotoxicity in kidneys from male and female mice treated with KPT-9274, characterized by acute tubular necrosis, tubular atrophy and regenerative hyperplasia. **C** Precent area with renal injury is estimated for treated NSG mice. **D** Representative images of TUNEL staining kidney sections and quantification of TUNEL^+^ cells in treated NSG mice. *p-value < 0.05. **E** Serum levels of BUN and creatinine and **F** erythropoietin (EPO) are measured in treated NSG and NRGS mice. *p-value < 0.05; **p-value < 0.01; ****p-value < 0.0001. **G** The relationship between EPO levels and the severity of renal injury (% renal cortex affected) in NSG mice. **H** RBC concentrations are measured in treated NSG and NRGS mice. *p-value < 0.05. **I** KPT-9274 exposure inhibits in vivo bone marrow erythropoiesis. The frequencies of erythroblast subsets (ProE, EryA, EryB and EryC) in treated bone marrow of NRGS mice are measured by CD71 and Ter119 staining. Data are expressed as mean ± SEM. *p-value < 0.05; **p-value < 0.01; ***p-value < 0.001. **J** The levels of SIRT3, acetyl-SOD, full length PARP1, cleaved PARP1, cleaved Caspase-3 and cleaved Caspase-7 after treatment of KPT-9274 at various concentrations in IMCD3 cells as detected by Western blotting. Results are representative of 2 replicates. **K** ROS levels in IMCD3 cells after treatment of KPT-9274 at various concentrations as measured by CM-H_2_DCFDA flow cytometric analysis; *p-value < 0.05; **p-value < 0.01. **L** IMCD3 cell apoptosis being treated with increasing concentrations of KPT-9274 and rescued by SIRT3 activator Honokiol as measured by Annexin V/PI staining. *p-value < 0.05; **p-value < 0.01. *ns* not significant

Anemia and kidney injury have been linked to erythropoietin (EPO) production deficiency. Female KPT-9274-treated mice had consistently lower levels of EPO (Fig. 1F and Additional file 1: Figure S2), which were inversely correlated with increased severities of kidney injury (Fig. 1G). Concomitantly, the number of red blood cells and other CBC parameters (HCT, HBG, MCV, MCH and reticulocyte) were reduced in female mice, suggestive of anemia associated with KPT-9274 treatment (Fig. 1H and Additional file 1: Figure S3). KPT-9274 treatment resulted in lower percentage of late stage Ter119^high^ erythroblasts and rise of ProE cells in bone marrow of female mice (Fig. 1I and Additional file 1: Figure S4). Within Ter119^high^ population, the frequency of late basophilic and polychromatic erythroblasts (EryB) was increased and concomitantly orthochromatic erythroblasts (EryC) was markedly reduced, suggesting inhibition of erythroblast differentiation and production of mature erythroblast subsets.

Sirtuin-3 (SIRT3) is a NAD^+^-dependent lysine deacetylase that participates in mitochondrial respiration. SIRT3 is also implicated in renal function through the regulation of reactive oxygen species (ROS). In IMCD3 cells treated with KPT-9274, we observed a dose-dependent decrease in SIRT3 expression and a concomitant rise in acetyl-manganese superoxide dismutase (Fig. 1J) and ROS production (Fig. 1K). Additionally, KPT-9274 treatment caused PARP1/caspase-3/caspase-7 cleavage and enhanced the frequency of apoptotic cells which can be rescued by SIRT3 activator, Honokiol (Fig. 1L). These results suggest that KPT-9274-induced nephrotoxicity results from the reduced activity of SIRT3.

The activation of the NAPRT1-depended salvage pathway through the supplementation of niacin [8] as an alternative way for NAD^+^ production, has been shown to circumvent the toxicity seen with NAMPT inhibitors. Tumor-specific promoter hypermethylation and loss of *NAPRT1* protein expression have also been observed in subtypes of lung, pancreatic, and ovarian cancers [8], making these tumors highly dependent on NAMPT for NAD^+^ production. We showed that co-administration of 30 mg/kg of niacin orally decreased the magnitude of renal lesions without affecting serum levels of creatinine and BUN in KPT-9274-treated NSG mice (Fig. 2A–C) and rescued the decreased EPO levels caused by KPT-9274 (Fig. 2D). KPT-9274 maintained its efficacy toward NAPRT1-negative AML cells with niacin supplement (Fig. 2E).

**Fig. 2:**
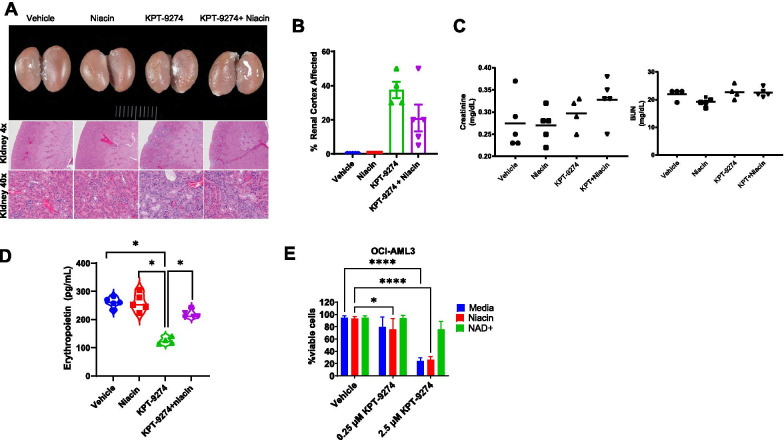
Niacin supplement rescues KPT-9274-induced acute kidney toxicity. **A** Gross images (top panel) and H&E staining (middle and bottom panels) of renal lesions in NSG mice which received KPT-9274 treatment for 3 weeks. Mice treated with KPT-9274 show marked pitting on the renal surface, which corresponds histologically to the areas of tubular collapse and fibrosis. The pitting is ameliorated by niacin treatment. **B** Percent areas with renal injury are quantified for each treatment group. **C** Serum levels of creatinine and BUN are measured in treated NSG mice. Levels across all groups are within normal limits with a slight increase in creatinine levels in KPT-9274-treated mice. **D** Measurement of erythropoietin in the serum of KPT-9274 + niacin-, KPT-9274-, niacin-, and vehicle-treated NSG mice after 3 weeks of treatment.*p-value < 0.05. **E** KPT-9274 maintains potency toward AML cells in the presence of niacin. OCI-AML3 cells were treated with vehicle, 0.25 µM KPT-9274 or 2.5 µM KPT-9274 in the presence of normal media, niacin or NAD + for 48 h before being subject to Annexin V/PI staining and flow cytometry analysis. Data from three independent experiments are expressed as mean ± SEM. *p-value < 0.05; ****p-value < 0.0001

In conclusion, our study delineated the mechanism of KPT-9274-mediated toxicity and sheds light onto developing strategies to improve the tolerability of this important anti-AML inhibitor. Our data also reaffirm that stratifying patients by NAMPT and NAPRT1 levels may determine the patient population that will benefit the most from NAMPT inhibition.

## Supplementary Information

**Additional file 1**. Supplementary Table I and Supplementary Figures.

**Additional file 2**. Supplementary Methods.

## Data Availability

Not applicable.

## Abbreviations

NAMPT: Nicotinamide phosphoribosyltransferase
EPO: Erythropoietin
NAD: Nicotinamide dinucleotide
GI: Gastrointestinal
BUN: Blood-urea-nitrogen
MnSOD: Acetyl-manganese superoxide dismutase
ROS: Reactive oxygen species
AML: Acute myeloid leukemia
