# Supplementary material for: Anti-tumor NAMPT inhibitor, KPT-9274, mediates gender-dependent murine anemia and nephrotoxicity by regulating SIRT3-mediated SOD deacetylation
Source: J Hematol Oncol. 2021 Jun 29;14:101. doi: 10.1186/s13045-021-01107-0 (PMC8243474; doi:10.1186/s13045-021-01107-0)
Supplement: Supplementary file 1 — Additional file 1. Supplementary Table I and Supplementary Figures. [file 13045_2021_1107_MOESM1_ESM.pdf]

Supplementary Table I. Key electrolytes measured in sera of NSG mice being treated with vehicle or KPT-9274

	Male		Female	
	Vehicle	KPT-9274	Vehicle	KPT-9274
Sodium (mmol)	149.2±0.6	149.2±0.5	148.7±0.9	144.6±0.05*
Potassium (mmol)	4.6±0.1	4.6±0.07	4.5±0.2	4.8±0.2
Chloride (mmol)	114.3±0.5	114.6±0.5	114.6±0.8	115.8±1.3

*p<0.05

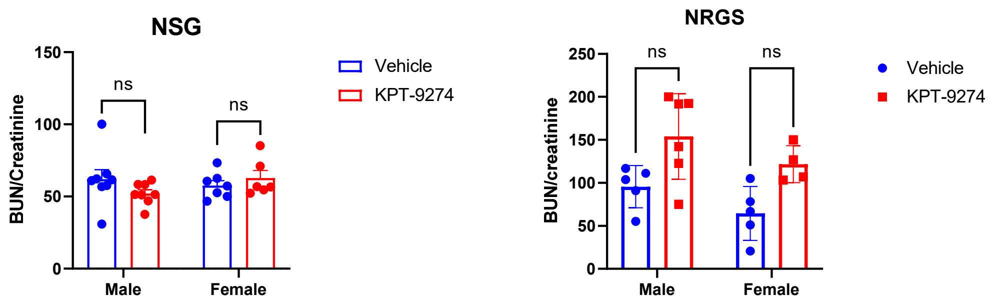

Supplementary Figure S1. BUN/creatinine ratios in NSG and NRGS mice treated with vehicle or 150 mg/kg KPT-9274. Data are expressed as mean±SEM.

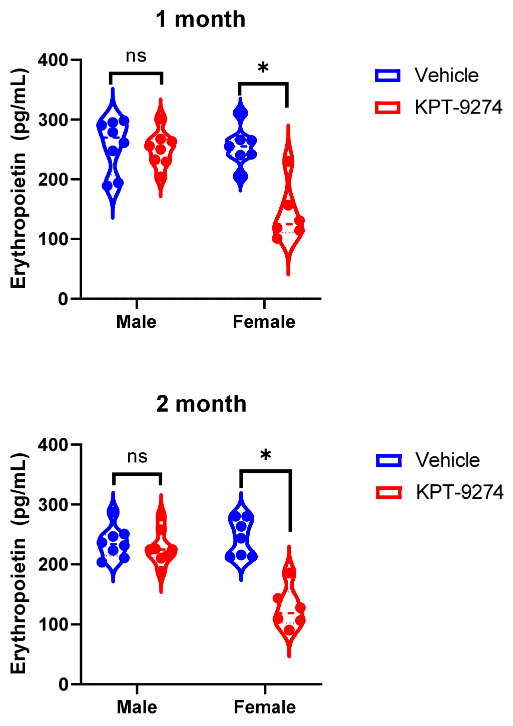

Supplementary Figure S2. EPO levels in male and female NSG mice which received either vehicle or KPT-9274 for 1 and 2 months. *p-value<0.05.

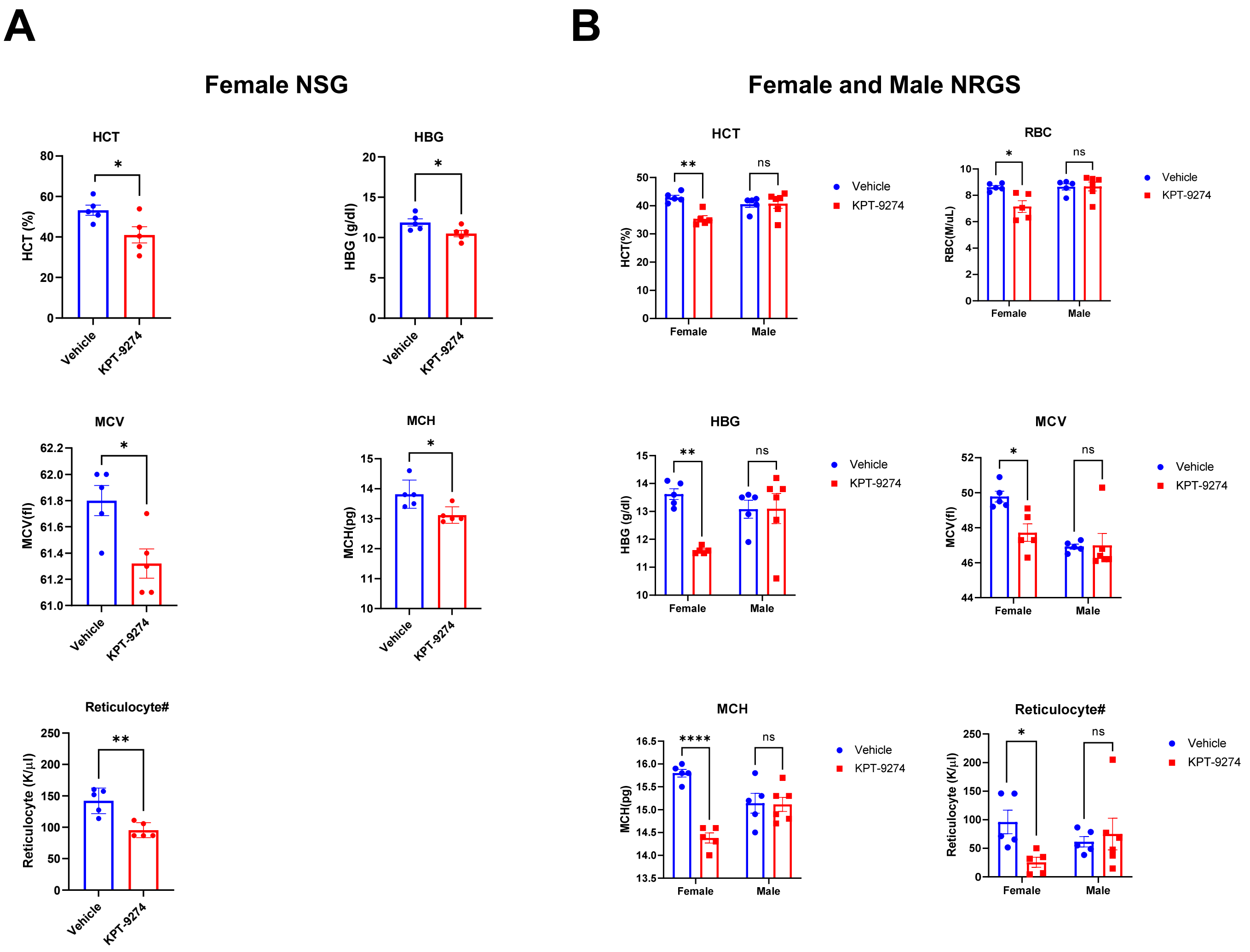

Supplementary Figure S3. KPT-9274 triggers gender-dependent anemia in NSG and NRGS mice. HCT, RBC, HBG, MCV, MCH and reticulocyte concentration of (A) female NSG and (B) NRGS mice of both genders which received vehicle or KPT-9274 are measured. Data are expressed as mean±SEM. *p<0.05; **p<0.01;****p<0.0001.

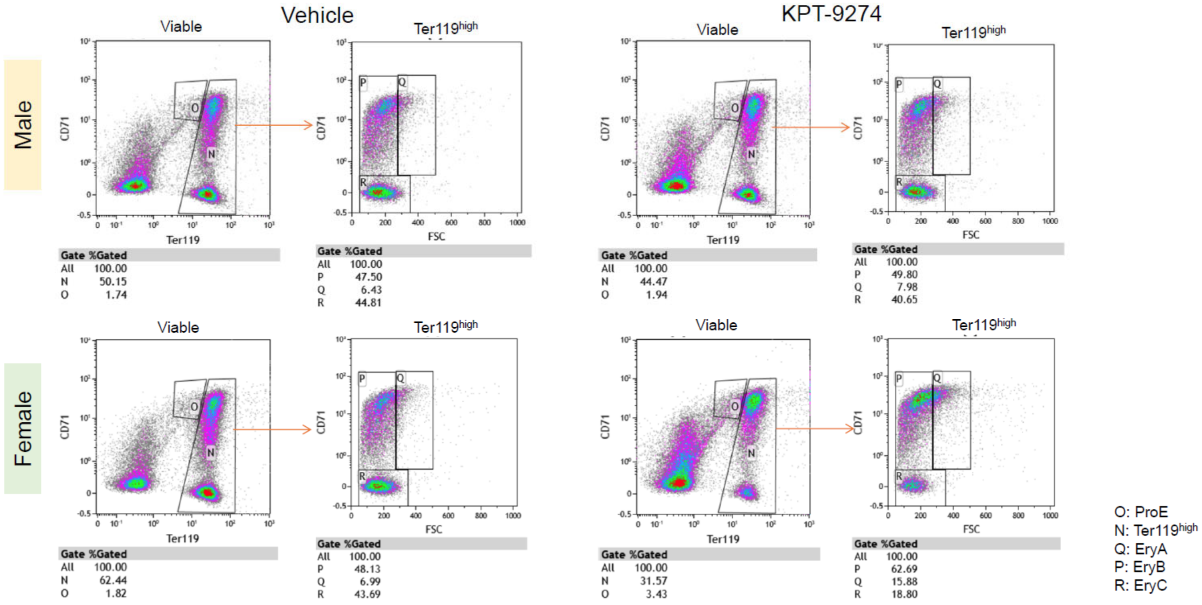

Supplementary Figure S4. Representative flow cytometry dotplot depicting the effect of KPT-9274 on the distributions of erythroblast subsets of the NRGS bone marrow as defined by surface expression of CD71 and Ter119.
