# Supplementary material for: Anti-tumor NAMPT inhibitor, KPT-9274, mediates gender-dependent murine anemia and nephrotoxicity by regulating SIRT3-mediated SOD deacetylation
Source: J Hematol Oncol. 2021 Jun 29;14:101. doi: 10.1186/s13045-021-01107-0 (PMC8243474; doi:10.1186/s13045-021-01107-0)
Supplement: Supplementary file 2 — Additional file 2. Supplementary Methods. [file 13045_2021_1107_MOESM2_ESM.docx]

**Methods**

*Drugs*

KPT-2974 was obtained from Selleckchem. Honokiol was purchased from Cayman chemicals, USA. Regarding the cell viability assay, different doses of Honokiol (0, 10 and 20μM) were incubated with IMCD3 cells for 48 hours in the presence of KPT-9274.

*Animal Studies*

Nonobese diabetic/severe combined immunodeficiency mice with IL2 receptor gamma chain mutation (NSG) mice were purchased from Jackson Laboratory. NOD.Cg-*Rag1^tm1Mom^* *Il2rg^tm1Wjl^* Tg(CMV-IL3,CSF2,KITLG)1Eav/J (NRGS) mice were routinely bred and maintained in OSU animal facility. Mice were 4 to 12 weeks old at the beginning of the experiment. Experiments were carried out under protocols approved by The Ohio State University Institutional Animal Care and Use Committee. Three independent *in vivo* studies were performed: 1) gender-dependent toxicity study of NSG mice, 2) gender-dependent toxicity study of NRGS mice, and 3) niacin rescue study of NSG mice. For studies 1) and 2), both female and male were used to assess gender-dependent toxicity. For study 3), only female mice were used. The endpoint for study 1) is 3 months of treatment and the endpoint for studies 2) and 3) is 3 weeks of treatment. For studies 1) and 2), mice were dosed via oral gavage with vehicle or KPT-9274 (150 mg/kg once daily). For study 3), mice received vehicle, niacin alone (30mg/kg MWF), KPT-9274 alone (150 mg/kg once daily) or a combination of niacin and KPT-9274. At endpoints of studies, all mice were euthanized and further analyzed for treatment effects.

*Histopathology*

Kidneys, eyes, and representative sections of stomach, jejunum and large intestine were fixed in 10% neutral buffered formalin, paraffin embedded, sectioned at 5uM onto glass slides and stained with hematoxyline and eosin (H&E) by routine methodology. Bones were decalcified in Surgical I formic acid and stained for H&E.

*Erythropoietin protein quantification analysis*

Whole blood was collected from mice treated with KPT-9274, niacin, KPT-9274 coadministered with niacin and vehicle control. Plasma was collected by centrifuging samples for 20 minutes at 2000 x g and placed in separate tubes. EPO measurement was performed with Mouse EPO ELISA kit (RayBio, Peachtree Corners, GA) and analyzed according to the manufacture’s protocol. Plate was read using a DTX plate reader (Labsystems mulitskan MCC1340) at 450nM and 540nM wavelengths.

*Serum analysis (blood urea nitrogen and creatinine), electrolyte levels and complete blood count*

Serum BUN, creatinine, electrolytes, CBC (RBC, HBG, HCT, MCV, MCH and reticulocytes) were measured at **Comparative Pathology & Mouse Phenotyping Shared Resource** of Ohio State University.

*Cell line culture conditions*

Cell line IMCD-3 (mouse inner medullary collecting duct-3) and OCI-AML3 were purchased from ATCC (Manassas, VA). Cell lines were cultured in recommended media conditions from vendors with the addition of 10 000 U of penicillin, 10 mg of streptomycin, and 200 mM of glutamate. All cells were kept in a 37°C, 5% carbon dioxide incubator.

*Immunoblotting*

IMCD3 cells were harvested and washed. Cells were lysed using a cell lysis buffer supplemented with protease inhibitors. Lysates were quantified using a BCA assay and proteins were separated using SDS-PAGE. Western blotting was performed by transferring proteins onto nitrocellulose paper and proteins were probed with anti-SIRT3 (Cell Signaling Technology, Inc., Danvers, MA) and anti-Actin (Santa Cruz Biotechnology, Santa Cruz, CA). Blots were washed and primary antibodies were probed with HRP-conjugated anti-mouse or –goat secondary antibodies. Blots were developed using Advansta WesternBright ECL.

*Flow cytometry measurement of erythroblast subpopulations*

Bone marrow cells were isolated from femur bones. Erythroblast differentiation was evaluated using CD71 and Ter119 surface marker expression and cell size characteristics (early erythroblasts (ProE), CD71^high^Ter119^low^; basophilic erythroblasts (EryA), CD71^high^Ter119^high^FSC^high^; late basophilic and polychromatic erythroblasts (EryB), CD71^high^Ter119^high^FSC^low^; and orthochromatic erythroblasts (EryC), CD71^low^Ter119^high^FSC^low^) as previously described^1^. Cells were incubated with Fc receptor blocker for 5 min before being incubated with CD71-PE and Ter119-FITC (BDbioscience) on ice in the dark for 30 min. Samples were washed twice and resuspended in DPBS with 2% FBS prior to analysis using a BD LSRFortessa flow cytometer.

*TUNEL assay*

Kidneys were fixed with 10% formalin and subsequently embedded in paraffin. Then, kidneys were sectioned and subject to TUNEL staining. TUNEL staining was conducted using an ApopTag Peroxidase In Situ Apoptosis Detection Kit (Sigma-Aldrich) according to the manufacturer’s protocol. For each paraffin section, 3 fields were randomly selected and the number of TUNEL-positive cells was evaluated at ×40 magnification.

*Statistical analysis*

Statistical analyses were performed using Prism 9.0 software. Statistical significance of differences was determined by Mann-Whitney U test, Student’s t-test or analysis of variance (ANOVA). Tukey’s test was used for *post hoc* analysis for ANOVA. To assess the normal distribution and equal variance of the data, Kolmogorov-Smirnov and Levene tests were employed. Probability values of *p*<0.05 and *p*<0.01 were chosen as statistical significance.

Reference:

1. Koulnis, M. *et al.* Identification and Analysis of Mouse Erythroid Progenitors using the CD71/TER119 Flow-cytometric Assay. *Jove-J Vis Exp* (2011).
